# Generation of An Endogenous FGFR2–BICC1 Gene Fusion/58 Megabase Inversion Using Single-Plasmid CRISPR/Cas9 Editing in Biliary Cells

**DOI:** 10.3390/ijms21072460

**Published:** 2020-04-02

**Authors:** Andreas Reicher, Antoneicka L Harris, Felix Prinz, Tobias Kiesslich, Miaoyan Wei, Rupert Öllinger, Roland Rad, Martin Pichler, Lawrence N Kwong

**Affiliations:** 1 Division of Oncology, Medical University of Graz, Graz 8036, Austria; AReicher@cemm.oeaw.ac.at (A.R.); felix.prinz@medunigraz.at (F.P.); 2 Research Unit for Non-Coding RNA and Genome Editing, Medical University of Graz, Graz 8036, Austria; 3 Department of Translational Molecular Pathology, The University of Texas MD Anderson Cancer Center, Houston, TX 77030, USA; ALHarris3@mdanderson.org (A.L.H.); miaoyan-wei@sjtu.edu.cn (M.W.); 4 Institute for Physiology and Pathophysiology, Paracelsus Medical University, Salzburg 5020, Austria; tobias.kiesslich@pmu.ac.at; 5 Department of General Surgery, Shanghai Jiao Tong University, Shanghai 200240, China; 6 Institute of Molecular Oncology and Functional Genomics, School of Medicine, Technische Universität München, Munich 81675, Germanyroland.rad@tum.de (R.R.)

**Keywords:** CRISPR, cholangiocarcinoma, *FGFR2*, fusion, inversion

## Abstract

Fibroblast growth factor receptor 2 (*FGFR2*) gene fusions are *bona fide* oncogenic drivers in 10–15% of intrahepatic cholangiocarcinoma (CCA), yet currently there are no cell lines publically available to study endogenous *FGFR2* gene fusions. The ability of clustered regularly interspaced short palindromic repeats (CRISPR)/Cas9 to generate large yet precise chromosomal rearrangements has presented the possibility of engineering endogenous gene fusions for downstream studies. In this technical report, we describe the generation of an endogenous *FGFR2*–Bicaudal family RNA binding protein 1 (*BICC1*) fusion in multiple independent cholangiocarcinoma and immortalized liver cell lines using CRISPR. *BICC1* is the most common *FGFR2* fusion partner in CCA, and the fusion arises as a consequence of a 58-megabase-sized inversion on chromosome 10. We replicated this inversion to generate a fusion product that is identical to that seen in many human CCA. Our results demonstrate the feasibility of generating large megabase-scale inversions that faithfully reproduce human cancer aberrations.

## 1. Introduction

Cholangiocarcinoma (CCA) is a relatively rare and aggressive cancer arising from epithelial cells of the biliary tree. Fibroblast growth factor receptor 2 (*FGFR2*) gene fusions are present in 10–15% of intra-hepatic CCA [1,2,3], wherein the 5′-portion of the *FGFR2* gene, including its kinase domain, is fused to different 3′-fusion partners. The most common partner genes include Periphilin 1 (*PPHLN1*), Bicaudal family RNA binding protein 1 (*BICC1*), Adenosylhomocysteinase Like 1 (*AHCYL1*), and Transforming Acidic Coiled-Coil Containing Protein 3 (*TACC3*) [4]. These partners facilitate oligomerization of the receptor independent of ligand-binding, resulting in a constitutively active kinase [1]. Despite the fusions’ bona fide oncogenic driver status in CCA, there is currently no CCA cell line publically available that has an endogenous *FGFR2* gene fusion, only cell lines with ectopic overexpression. Such overexpression has been done in the kinase-dependent Ba/F3 mouse B or the NIH373 mouse embryo fibroblast cell lines that do not necessarily resemble the molecular features of human CCA cells [1,5]. Moreover, ectopic overexpression often reaches supraphysiological levels.

To create endogenous *FGFR2* fusion-bearing cells, we have used the CRISPR/Cas9 system, which only requires the expression of Cas9 and target-specific sgRNAs [6] to induce concomitant DNA double strand breaks at the two fusion partners. Previously, this approach has been successfully used to model various genomic rearrangements, including translocations; deletions; and inversions that result in the formation of cancer-associated gene fusions such as *EML-ALK4, CD74-ROS1, KIF5B-RET, FIP1L1-PDGFRA, MLL-ENL,* or *EWSR1-FLI1* [7,8,9,10,11]. Here, we report the successful generation of the FGFR2–BICC1 gene fusion via a large, 58-megabase inversion using a single plasmid. 

## 2. Results

**Figure 1:**
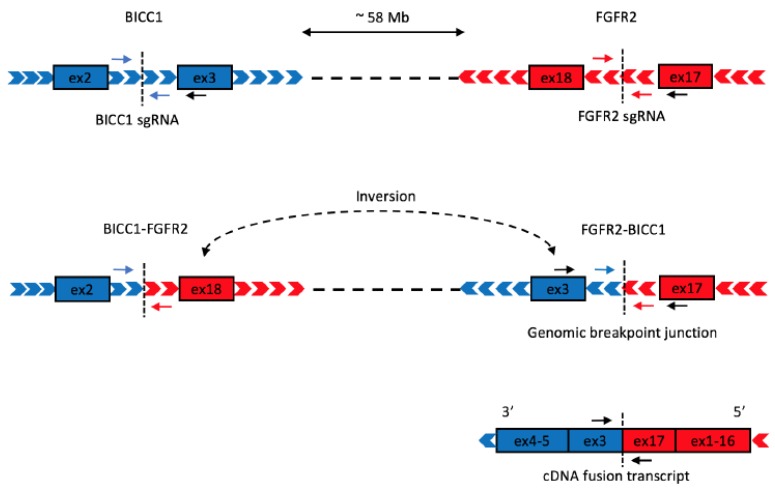
Schematic of clustered regularly interspaced short palindromic repeats (CRISPR)-induced inversion resulting in the fibroblast growth factor receptor 2 (FGFR2)–Bicaudal family RNA binding protein 1 (BICC1) gene fusion. (**A**) sgRNAs target the intronic region of *FGFR2* and *BICC1* on chromosome 10 (dotted lines). Arrows: primers for detection of WT *FGFR2*, WT *BICC1*, and *FGFR2–BICC1*. (**B**) The *FGFR2–BICC1* gene fusion results from an inversion of a 58-megabase fragment. (**C**) The fusion transcript comprises *FGFR2* exons 1-17 fused to *BICC1* exons 3-5.

**Figure 2:**
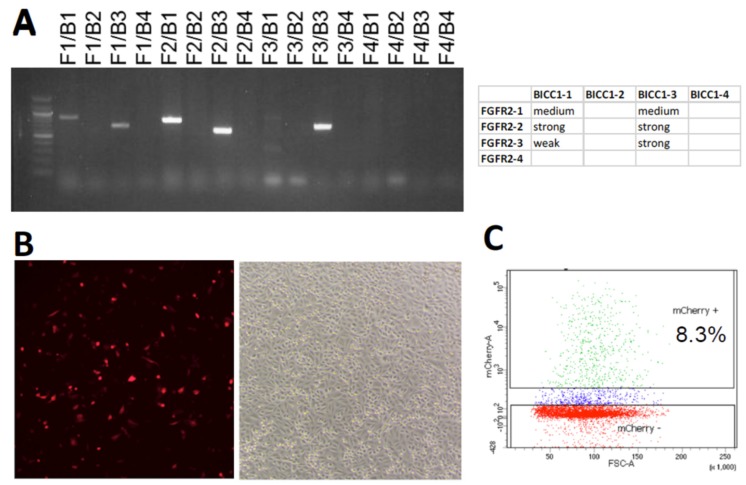
Discovery of sgRNA combinations that induce the *FGFR2–BICC1* fusion. (**A**) Detection of *FGFR2–BICC1* gene fusions using PCR after cells were transiently transfected with the two-plasmid system. (**B**) mCherry positivity (*left*) indicating transfection efficiency of cells (brightfield, *right*) transfected with the two-plasmid system 48 h after transfection. Magnification = 4×. (**C**) Representative FACS data of transfected cells 48h after transfection.

The *FGFR2–BICC1* fusion is the result of an inversion of a 58-megabase fragment on chromosome 10 (Figure 1). We first used the immortalized human hepatocyte cell line Hc3716-hTERT (hereafter “Hc3716”) [12] to replicate this inversion using CRISPR engineering, as hepatocytes are a known cell of origin for CCA [13]. In this strategy, one sgRNA targets intron 2 or 16 of *BICC1* and another targets intron 17 of *FGFR2*, stimulating DNA DSBs at both sites. We tested 4 sgRNAs for each gene, after they were individually cloned into an mCherry- and Cas9-expressing pX330 plasmid [14]. Cells were transiently transfected with all 16 possible combinations of one *FGFR2* sgRNA and one *BICC1* sgRNA. Using polymerase chain reaction (PCR) and primers designed to flank the genomic breakpoint junctions, 6 of the sgRNA combinations were found to efficiently introduce a genomic inversion resulting in an *FGFR2–BICC1* fusion (Figure 2a). The transfection efficiency was ~8% as measured by mCherry positivity (Figure 2b). Fluorescence-activated cell sorting (FACS) sorting was then used to enrich for mCherry-positive cells to increase the probability of identifying the correct inversion (Figure 2c).

**Figure 3:**
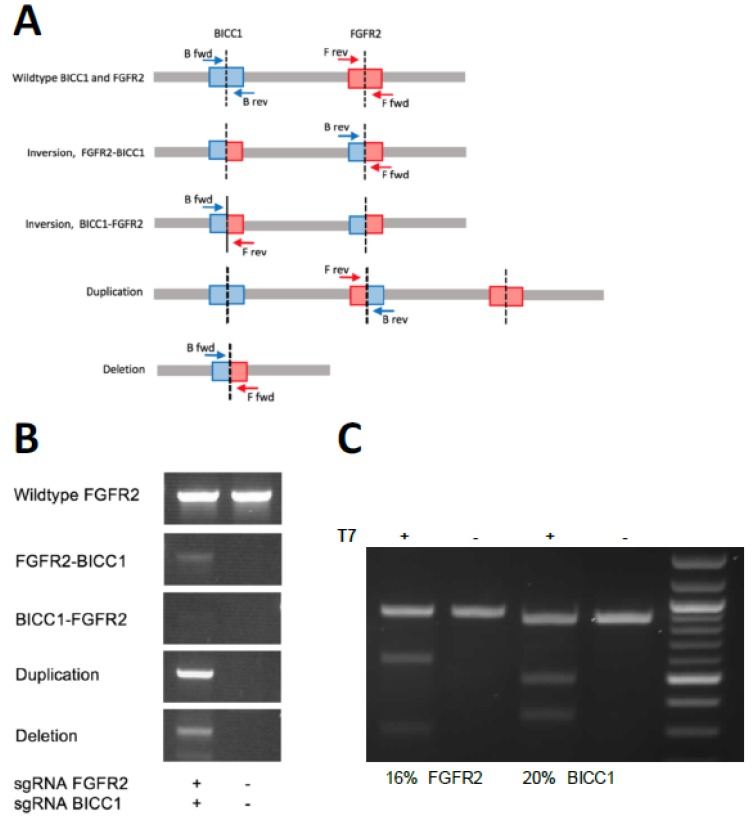
Detection of distinct genomic outcomes from sgRNA cutting. (**A**) Schematic of possible genomic outcomes of dual sgRNA cutting. (**B**) Detection of different genomic rearrangements in cells transfected with the two-plasmid system. (**C**) CRISPR editing efficiency after FACS sorting, as determined by T7 endonuclease assay. The indel frequencies are indicated below.

The sgRNA combination sgFGFR2-2/sgBICC1-1 (lane 5 in Figure 2a) was selected for further experiments, as it was among the three pairs with the most robust fusion generation. First, we determined if this sgRNA combination is also capable of inducing other genomic rearrangements other than the intended inversion (Figure 3a). As expected, we also detected the generation of a deletion between the two breakpoints as well as a duplication of the fragment resulting from a translocation event that can happen when the *FGFR2* locus is cut on one of the two chromosomes and BICC1 is cut on the other chromosome of a diploid cell. We also detected the other end of the inversion, namely, the *BICC1–FGFR2* fusion (Figure 3b). Next, we assessed the CRISPR editing efficiency of the two sgRNAs by the T7 endonuclease assay. Editing efficiency in FACS-sorted cells was 16% for the *FGFR2* locus and 20% for the BICC1 locus (Figure 3c). Clonal cell lines were then isolated that were positive for the *FGFR2–BICC1* gene fusion using a limiting dilution of approximately one cell per well. After 2–3 weeks of clonal expansion, we screened 47 clonal cell lines and detected the *FGFR2–BICC1* fusion using PCR in 2 clones (named 6 and 8). 

We next asked whether the system can be simplified by placing both sgRNAs on a single plasmid, with each sgRNA under the control of its own U6 promoter. Using this multiplex plasmid, we screened another 66 Hc3716 clones and again obtained two clones (named 2 and 4) with *FGFR2–BICC1* gene fusions. To further validate the plasmid and to facilitate future functional analyses, we used the multiplex plasmid to transfect the Hc3716-shp53 subline, in which TP53 is knocked down. We screened 70 clones and obtained one clone positive for the *FGFR2–BICC1* gene fusion. These results indicate that the single multiplex plasmid works equally as well as the two-plasmid system in vitro (Table 1). 

**Figure 4:**
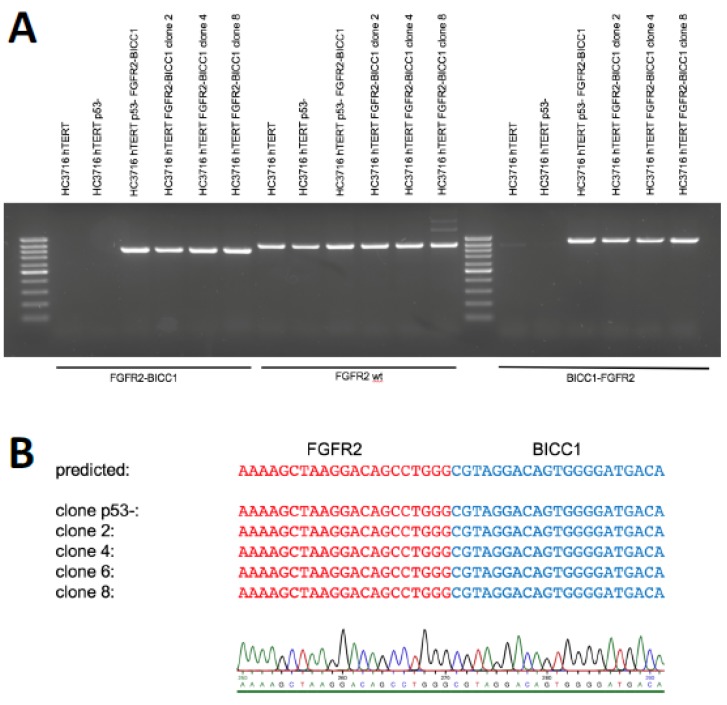
DNA validation of the genomic breakpoint junction of the inversion. (**A**) DNA was isolated from the clonal cell lines, and the *FGFR2–BICC1* and *BICC1-FGFR2* breakpoint junctions were detected PCR. The *FGFR2* WT allele around the CRISPR targeting site was amplified as a control. (**B**) Sanger sequencing of the genomic breakpoint junction.

We then assessed the Hc3716 clones together, whether they were produced by the two- or one-plasmid system. In three clones assessed by PCR (2, 4, and 8), both the *FGFR2–BICC1* fusion and the other end of the inversion, the *BICC1–FGFR2* fusion, were detected as expected (Figure 4a). Additionally, all clones still had a WT *FGFR2* allele. Sanger sequencing of the *FGFR2–BICC1* genomic breakpoint junction of all five clones revealed that the junction exactly matched the predicted sequence based on the CRISPR-induced double strand breaks (Figure 4b), without any gain or loss of sequence information. 

**Figure 5:**
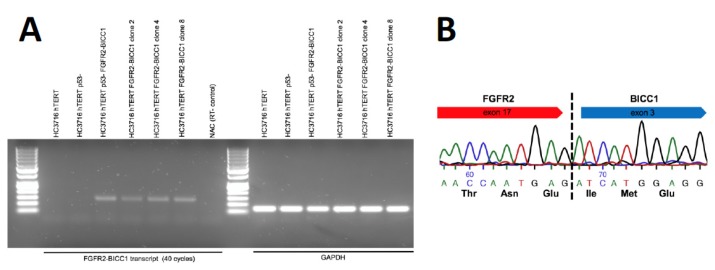
RNA validation of the *FGFR2–BICC1* fusion transcript (**A**) RNA was isolated from the clonal cell lines and reverse transcribed. The cDNA was used to detect the *FGFR2–BICC1* fusion using forward and reverse primers that map to exon 17 of *FGFR2* and exon3 of *BICC1*, respectively. *GAPDH* was used as a control. (**B**) Sanger sequencing of the exon–exon junction showing an in-frame fusion transcript.

We next asked whether the *FGFR2–BICC1* fusion is expressed in our clonal cell lines, and were able to detect the transcript by RT-PCR (Figure 5a). We sequenced the exon–exon junction, which demonstrated that exon 17 of *FGFR2* is fused in frame to exon 3 of *BICC1* (Figure 5b). This exon–exon junction is identical to the exon–exon junction found in human tumors expressing an *FGFR2–BICC1* fusion [1]. Additionally, the sequence shows that the transcript is spliced correctly without error in our clonal cell lines.

To confirm the broad applicability of our single-plasmid CRISPR fusion approach, we applied it to three additional cell lines: two intrahepatic, *FGFR2*-fusion-negative CCA cell lines HUH-28 and CCSW-1, and the immortalized cholangiocyte cell line MMNK-1. As above, individual flow-sorted, mCherry-positive, limiting-diluted clones were screened by PCR to identify *FGFR2–BICC1* fusions. For HUH-28, five positive clones were identified out of 32; for MMNK-1, two positive clones out of 32; and for CCSW-1, 15 positive clones out of 155 (Table 1 and Supplementary Figure S1). Next, we validated 1/3 HUH-28 and 2/2 MMNK-1 clones as expressing the *FGFR2–BICC1* RNA by RT-PCR (Supplementary Figure S2). Interestingly, two HUH-28 fusion-positive clones did not express the transcript, and we speculate that a known *BICC1* frameshift mutation present in that cell line may lead to nonsense-mediated decay. Thus, it is imperative to further validate DNA-positive clones. Overall, these results confirm that our single-plasmid construct can be used to efficiently induce bona fide *FGFR2–BICC1* fusions in a variety of human cell lines.

## 3. Discussion

In the present study, we have successfully generated an endogenous *FGFR2–BICC1* gene fusion in multiple human cell lines using the CRISPR-Cas9 system. To our knowledge, the 58-megabase inversion is the largest inversion or deletion event that has ever been artificially generated in human cells using the CRISPR/Cas9 system. While kilobase-sized deletions or inversions are generated in cells with high frequency, the efficiency decreases with increasing fragment size [15]. The largest deletion reported in the literature is a 30-megabase deletion that was generated in a near-haploid cell line to obtain the first fully haploid cell [16]. Moreover, our straightforward fusion generation system can potentially facilitate future efforts to preclinically interrogate *FGFR2–BICC1* functions in parallel with ongoing clinical trials assessing FGFR inhibitors in fusion-positive CCA [17]. Currently, we are assessing the phenotype of the fusion-positive cells.

## 4. Materials and Methods

### 4.1. Construction of CRISPR Plasmids

CRISPR/Cas9 target sites were selected using Benchling gRNA design tool and were cloned into the pX330 plasmid expressing the gRNA under the control of an U6 promoter, a Cas9 expression cassette, and mCherry as a selection marker. To obtain the FGFR2–BICC1 fusion we designed four sgRNAs located in the intron between exon 17 and 18 of the FGFR2 gene, three sgRNAs located in the intron between exon 2 and exon 3 of *BICC1,* and one sgRNA located in the intron between exon 16 and 17 of *BICC1*. The gRNA sequences are provided in Supplementary Table S1. gRNA sequences were individually cloned into the pX330 plasmid using the *BbsI* restriction site as described by Ran et al. [18]. Briefly, synthesized top and bottom oligos were annealed and phosphorylated using T4 PNK and cloned into the pX330 plasmid in a ligation reaction containing *BbsI* and T4 ligase. The ligation reaction was incubated for 1h, treated with PlasmidSafe exonuclease and transformed into Stbl3 chemically competent E. coli cells. Cells were incubated overnight and colonies were picked for an overnight culture. Plasmid DNA was isolated using QIAprep Spin miniprep kit, and the plasmid sequence was verified by Sanger sequencing. For construction of the multiplex plasmid containing two sgRNAs in a single plasmid, the entire U6promoter_gRNA_terminator cassette from one plasmid (*FGFR2*, 2) was cloned into another other (*BICC1*, 1) using the *PciI* restriction site upstream of that cassette. The resulting multiplex plasmid thus contains both sgRNAs under the control of two independent U6 promoters.

### 4.2. Transfection

The immortalized hepatocyte cell line HC3716-hTERT was used for all experiments. Cells were transiently transfected with the pX330 plasmids using lipofectamine 3000 as described by the manufacturer. To enrich for transfected cells, mCherry positive cells were obtained using fluorescence-activated cell sorting (FACS) 48 h after transfection.

### 4.3. Isolation of Single Cells

Limiting dilution was used to obtain clonal cell lines with the *FGFR2–BICC1* fusion. Cells were seeded in a 96-well plate with a concentration of 10 cells per ml, resulting in approximately 1 cell per well. Cells were expanded for 2–3 weeks, and wells containing multiple colonies and hence polyclonal cell lines were excluded.

### 4.4. Detection of Genomic Rearrangement by PCR

Either mixed cell populations obtained before limiting dilution or clonal cell lines were analyzed for the presence of genomic rearrangements by using PCR. DNA was isolated using a DNA isolation buffer containing Proteinase K, Tween, Triton-X, KCl, and (NH_4_)_2_SO_4_. Primers located on the *FGFR2* gene and the *BICC1* gene were used in different combinations to detect either WT *FGFR2*, WT *BICC1*, the *FGFR2–BICC1* fusion, the *BICC1–FGFR2* fusion, a deletion event between the two target sites, or a duplication. Primer combinations and primer sequences are listed in Supplementary Table S2 and S3. To determine the exact fusion breakpoint of the *FGFR2–BICC1* fusion, the PCR products of clonal cell lines were analyzed by Sanger sequencing. 

### 4.5. T7 Endonuclease Assay

The T7 endonuclease assay was used to determine genome targeting efficiency of different sgRNAs. T7 Endonuclease I from NEB was used, and the assay performed as described by the manufacturer. Briefly, Platinum SuperFi PCR Master Mix was used to amplify the targeted locus and the PCR product was purified using a PCR purification kit (QIAGEN). Purified PCR product was annealed in a thermocycler and subsequently incubated with T7 Endonuclease I. Reactions were analyzed on a gel, and the editing efficiency was calculated as described by Ran et al. [18].

### 4.6. RNA Isolation and RT-PCR 

To analyze the expression of the *FGFR2–BICC1* transcript, Trizol was used to isolate total RNA. QIAGEN OneStep RT-PCR kit was used to obtain cDNA, which was used in a subsequent PCR to detect the expression of the fusion transcript. The forward and reverse primers are located on exon 17 FGFR2 and on the exon 3 of BICC1, respectively, meaning that the PCR product spans the exon–exon junction of the fusion transcript. *GAPDH* was used as a control. Primer sequences are listed in Supplementary Table S2. The PCR product was sequenced to determine the exact sequence of the exon–exon junction.

## 5. Conclusions

Overall, our approach generated the *FGFR2–BICC1* fusion at a rate of 1–15% across four cell lines. We will make the dual-sgRNA plasmid available on Addgene for public use. We suggest that other gene fusions with *FGFR2* can also be readily created through adaptions of our CRISPR/Cas9 approach, as many of the fusion partners also reside on chromosome 10. It is clear that CRISPR technology is a powerful method for reproducing a wide range of human genetic aberrations, and our results demonstrate that even very large chromosome rearrangement events can be efficiently generated using a single multiplex plasmid.

## Figures and Tables

**Table 1:** *FGFR2–BICC1* Fusion Efficiency.

Cell Line	Plasmid System	# Colonies Screened	# Colonies Positive	% Colonies Positive	# +Colonies Expressing Fusion
Hc3716	2 plasmids	47	2	4.2%	2/2
Hc3716	1 plasmid	66	2	3.0%	2/2
Hc3716 shp53	1 plasmid	70	1	1.4%	1/1
HUH-28	1 plasmid	32	5	15%	1/3
MMNK-1	1 plasmid	32	2	6.2%	2/2
CCSW-1	1 plasmid	155	15	9.7%	ND

# = number. + = positive.

## Supplementary Materials

Supplementary materials can be found at https://www.mdpi.com/1422-0067/21/7/2460/s1. Table S1. sgRNA sequences. Table S2. Primer sequences. Table S3. Primer combinations. Figure S1. PCR identification of HUH-28, MMNK-1, and CCSW-1 fusion-positive clones. Figure S2. RT-PCR of Huh-28 and MMNK-1 fusion-positive and control clones.

## References

[1] Arai Y., Totoki Y., Hosoda F., Shirota T., Hama N., Nakamura H., Ojima H., Furuta K., Shimada K., Okusaka T. (2014). Fibroblast growth factor receptor 2 tyrosine kinase fusions define a unique molecular subtype of cholangiocarcinoma. Hepatology.

[2] Borad M.J., Gores G.J., Roberts L.R. (2015). Fibroblast growth factor receptor 2 fusions as a target for treating cholangiocarcinoma. Curr. Opin. Gastroenterol..

[3] Wu Y.M., Su F., Kalyana-Sundaram S., Khazanov N., Ateeq B., Cao X., Lonigro R.J., Vats P., Wang R., Lin S.F. (2013). Identification of Targetable FGFR Gene Fusions in Diverse Cancers. Cancer Discov..

[4] Li F., Peiris M.N., Donoghue D.J. (2019). Functions of FGFR2 corrupted by translocations in intrahepatic cholangiocarcinoma. Cytokine Growth Factor Rev..

[5] Tan L., Wang J., Tanizaki J., Huang Z., Aref A.R., Rusan M., Zhu S.J., Zhang Y., Ercan R., Liao R.G. (2014). Development of covalent inhibitors that can overcome resistance to first-generation FGFR kinase inhibitors. Proc. Natl. Acad. Sci. USA.

[6] Jinek M., Chylinski K., Fonfara I., Hauer M., Doudna J.A., Charpentier E. (2012). A programmable dual-RNA-guided DNA endonuclease in adaptive bacterial immunity. Science.

[7] Bempt M.V., Demeyer S., Mentens N., Geerdens E., De Bock C.E., Wlodarska I., Cools J. (2016). Generation of the Fip1l1–Pdgfra fusion gene using CRISPR/Cas genome editing. Nat. Publ. Group.

[8] Blasco R.B., Karaca E., Ambrogio C., Cheong T.C., Karayol E., Minero V.G., Chiarle R. (2014). Simple and Rapid In&nbsp; Vivo Generation of Chromosomal Rearrangements using CRISPR/Cas9 Technology. CellReports.

[9] Choi P.S., Meyerson M. (2014). Targeted genomic rearrangements using CRISPR/Cas technology. Nat. Commun..

[10] Reimer J., Knöß S., Labuhn M., Charpentier E.M., Göhring G., Schlegelberger B., Klusmann J.H., Heckl D. (2017). CRISPR-Cas9-induced t(11;19)/MLL-ENL translocations initiate leukemia in human hematopoietic progenitor cells in vivo. Haematologica.

[11] Torres R., Martin M.C., Garcia A., Cigudosa J.C., Ramirez J.C., Rodriguez-Perales S. (2014). Engineering human tumour-associated chromosomal translocations with the RNA-guided CRISPR-Cas9 system. Nat. Commun..

[12] Waki K., Anno K., Ono T., Ide T., Chayama K., Tahara H. (2010). Establishment of functional telomerase immortalized human hepatocytes and a hepatic stellate cell line for telomere-targeting anticancer drug development. Cancer Sci..

[13] Zhu Y., Kwong L.N. (2020). Insights into the origin of intrahepatic cholangiocarcinoma from mouse models. Hepatology.

[14] Van Chu T., Weber T., Wefers B., Wurst W., Sander S., Rajewsky K., Kühn R. (2015). Increasing the efficiency of homology-directed repair for CRISPR-Cas9-induced precise gene editing in mammalian cells. Nat. Biotechnol..

[15] Canver M.C., Bauer D.E., Dass A., Yien Y.Y., Chung J., Masuda T., Maeda T., Paw B.H., Orkin S.H. (2014). Characterization of genomic deletion efficiency mediated by clustered regularly interspaced short palindromic repeats (CRISPR)/Cas9 nuclease system in mammalian cells. J. Biol. Chem..

[16] Essletzbichler P., Konopka T., Santoro F., Chen D., Gapp B.V., Kralovics R., Brummelkamp T.R., Nijman S.M.B., Burckstummer T. (2014). Megabase-scale deletion using CRISPR/Cas9 to generate a fully haploid human cell line. Genome Res..

[17] Javle M., Lowery M., Shroff R.T., Weiss K.H., Springfeld C., Borad M.J., Ramanathan R.K., Goyal L., Sadeghi S., Macarulla T. (2018). Phase II Study of BGJ398 in Patients With FGFR-Altered Advanced Cholangiocarcinoma. J. Clin. Oncol. Off. J. Am. Soc. Clin. Oncol..

[18] Ran F.A., Hsu P.D., Wright J., Agarwala V., Scott D.A., Zhang F. (2013). Genome engineering using the CRISPR-Cas9 system. Nat. Protoc..

