# Supplementary material for: Generation of An Endogenous FGFR2–BICC1 Gene Fusion/58 Megabase Inversion Using Single-Plasmid CRISPR/Cas9 Editing in Biliary Cells
Source: Int J Mol Sci. 2020 Apr 2;21(7):2460. doi: 10.3390/ijms21072460 (PMC7178239; doi:10.3390/ijms21072460)
Supplement: Supplementary file 1 [file ijms-21-02460-s001.zip › ijms-742707-supp-original/supplementary-figure.docx]

**Supplementary Material**

**Figure S1.** PCR identification of HUH-28, MMNK-1, and CCSW-1 fusion-positive clones. Clones that failed validation by Sanger sequencing are not highlighted. For CCSW-1, a 3-primer set was used to detect WT (bottom band) and fusion (top band) FGFR2 simultaneously. pos = positive control.

**Figure S2.** RT-PCR of Huh-28 and MMNK-1 fusion-positive and control clones.
