# Supplementary material for: Generation of An Endogenous FGFR2–BICC1 Gene Fusion/58 Megabase Inversion Using Single-Plasmid CRISPR/Cas9 Editing in Biliary Cells
Source: Int J Mol Sci. 2020 Apr 2;21(7):2460. doi: 10.3390/ijms21072460 (PMC7178239; doi:10.3390/ijms21072460)
Supplement: Supplementary file 1 [file ijms-21-02460-s001.zip › ijms-742707-supp-original/supplementary-table.docx]

**Supplementary Material**

**Table S1.** sgRNA sequences.

| sgRNA | Target | Intron | Sequence |
| --- | --- | --- | --- |
| FGFR2-1 | FGFR2 | 17 | GTAATCCCAGCTACTCCGGG |
| FGFR2-2 | FGFR2 | 17 | GACCACGTCTGATGTACCCC |
| FGFR2-3 | FGFR2 | 17 | GAGATGTGGGTATTGGACGT |
| FGFR2-4 | FGFR2 | 17 | CAAGGTGAATACGGTTCGAG |
| BICC1-1 | BICC1 | 2 | GTATTAGTGTGCACAGCCGT |
| BICC1-2 | BICC1 | 2 | CTAATGCCGTGACCTAAAGG |
| BICC1-3 | BICC1 | 2 | CCGGTACGTAGCCAGTACTG |
| BICC1-4 | BICC1 | 16 | CCTCTTCATTTAGGTAAGCG |

**Table S2.** Primer sequences.

| Primer |  | Sequence |
| --- | --- | --- |
| FGFR2_1_fwd |  | TTCCCCTTCAATTATCCCCGAAA |
| FGFR2_1_rev |  | TCTCCTCCTGGGGAAGATTACA |
| FGFR2_2_fwd |  | CCTTCAGAAGTTGAAAGGAAAGGA |
| FGFR2_2_rev |  | CAGCATCCCAGAAGGGGAATG |
| BICC1_1_fwd |  | ACCCCCTGTAGAGACCTTGTG |
| BICC1_1_rev |  | ACATAGCATGGCACGTACTGA |
| BICC1_2_fwd |  | TTGCTCTGAGAAAAACAGCAAACA |
| BICC1_2_rev |  | CAGCAACGGAAATGGACGGT |
| BICC1_3_fwd |  | CCCTTGGTCCTTGTGGTAGG |
| BICC1_3_rev |  | TTGGTGGCTAATAGCTTCTTCTGT |
| Fusion transcript fwd |  | TGATGAGGGACTGTTGGCAT |
| Fusion transcript rev |  | TGGCCAAGCAATCTGCGTAT |

**Table S3.** Primer combinations.

| First Primer | Second Primer | Target |
| --- | --- | --- |
| FGFR2_1_fwd | BICC1_1_rev | FGFR2-BICC1 from sgRNAs F2/B1, F2/B2, F3/B1, F3/B2, F4/B1, F4/B2 |
| FGFR2_2_fwd | BICC1_1_rev | FGFR2-BICC1 from sgRNAs F1/B1, F1/B2 |
| FGFR2_2_fwd | BICC1_2_rev | FGFR2-BICC1 from sgRNAs F1/B3 |
| FGFR2_2_fwd | BICC1_3_rev | FGFR2-BICC1 from sgRNAs F1/B4 |
| FGFR2_1_fwd | BICC1_2_rev | FGFR2-BICC1 from sgRNAs F2/B3, F3/B3, F4/B3 |
| FGFR2_1_fwd | BICC1_3_rev | FGFR2-BICC1 from sgRNAs F2/B4, F3/B4, F4/B4 |
| BICC1_1_fwd | FGFR2_1_rev | BICC1-FGFR2 from sgRNAs F2/B1 |
| BICC1_1_fwd | FGFR2_1_fwd | Deletion from sgRNAs F2/B1 |
| FGFR2_1_rev | BICC1_1_rev | Duplication from sgRNAs F2/B1 |
| FGFR2_1_fwd | FGFR2_1_rev | FGFR2 locus cut by sgRNA F2 (T7 assay) |
| BICC1_1_fwd | BICC1_1_rev | BICC1 locus cut by sgRNA B1 (T7 assay) |
| Fusion Transcript fwd | Fusion transcript rev | Detection of FGFR2 fusion transcript, fwd primer maps to exon 17 of FGFR2 and the rev primer maps to exon 3 of BICC1 |
