# Supplementary figures and images for: Generation of An Endogenous FGFR2–BICC1 Gene Fusion/58 Megabase Inversion Using Single-Plasmid CRISPR/Cas9 Editing in Biliary Cells
Source: Int J Mol Sci. 2020 Apr 2;21(7):2460. doi: 10.3390/ijms21072460 (PMC7178239; doi:10.3390/ijms21072460)

Figure S1

**HUH-28 clones**

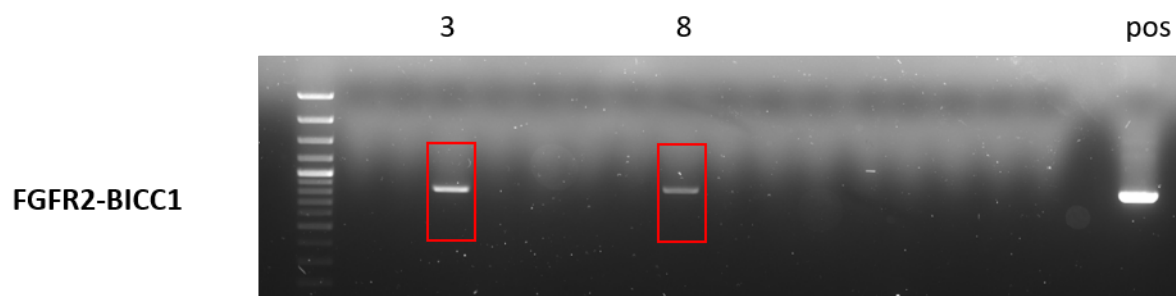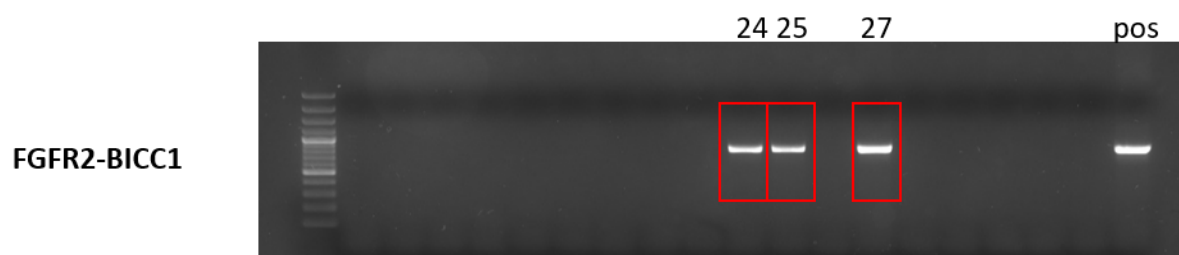

**MMNK-1 clones**

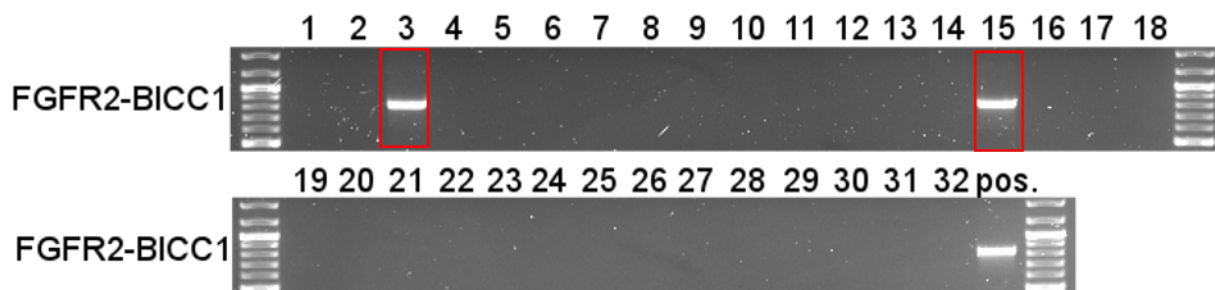

**CCSW-1 clones**

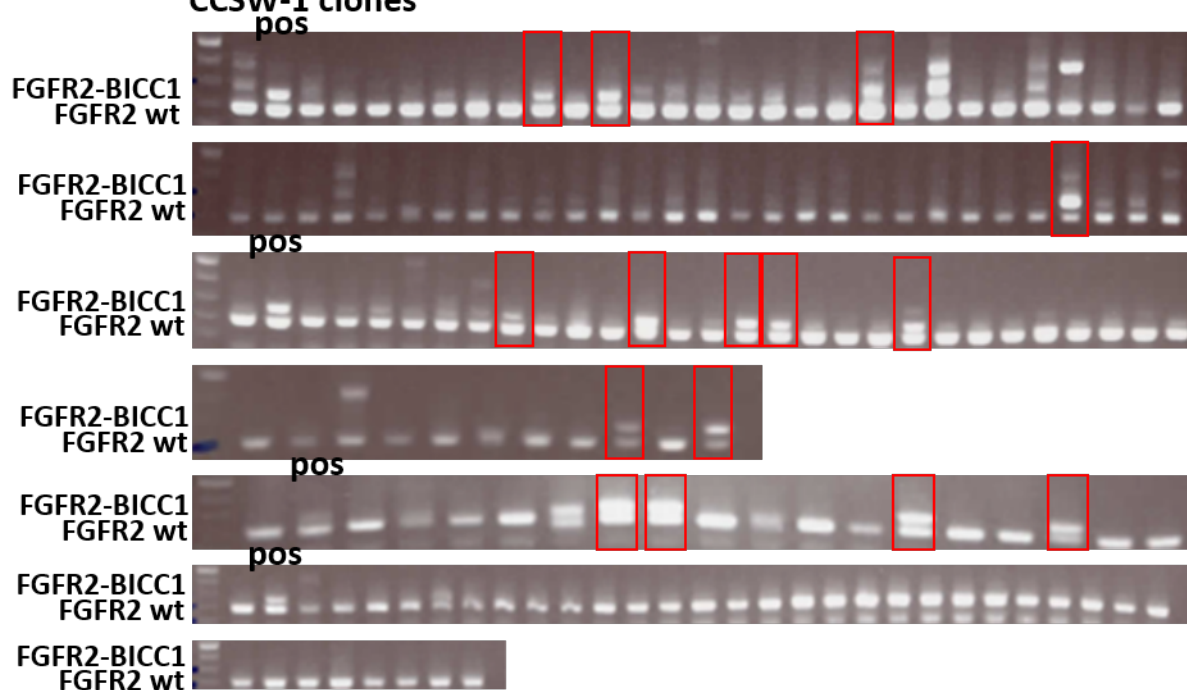

Supplement: Supplementary file 1 [file ijms-21-02460-s001.zip › ijms-742707-supp-original/FGFR2-BICC1 Figure S1.pdf]

Figure S2

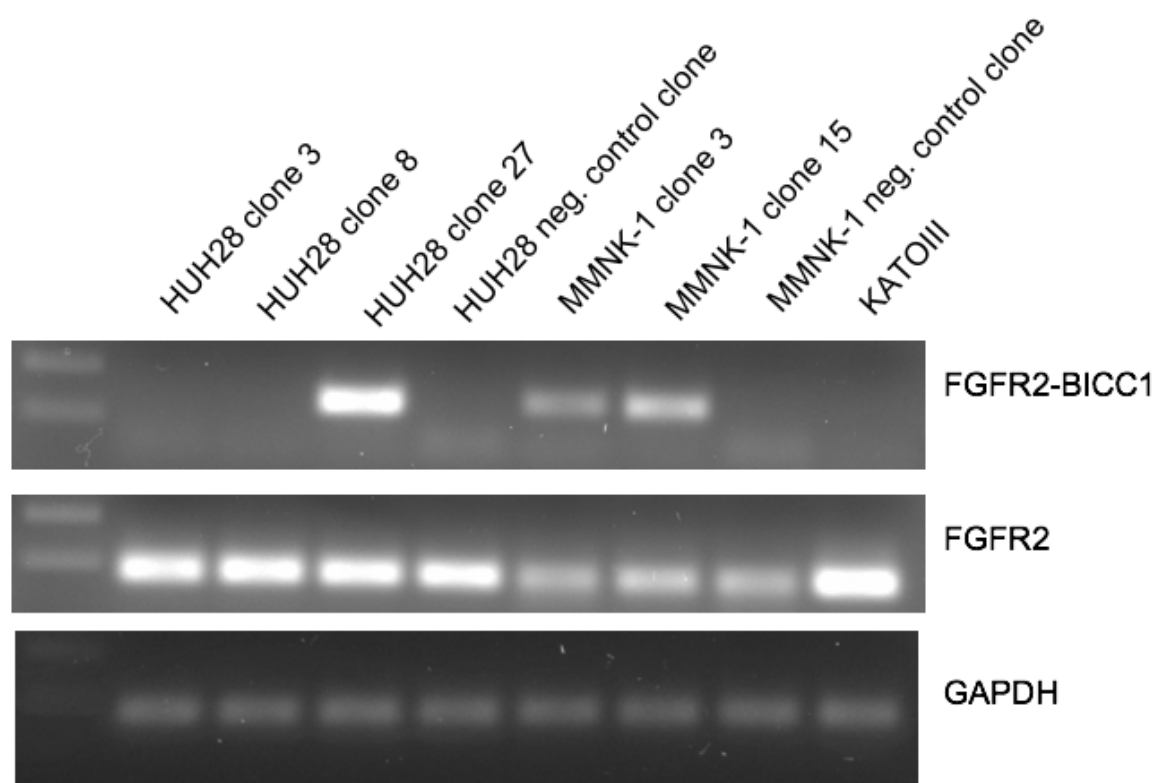

Supplement: Supplementary file 1 [file ijms-21-02460-s001.zip › ijms-742707-supp-original/FGFR2-BICC1 Figure S2.pdf]
